# Diversity of *Eimeria* (Apicomplexa: Eimeriidae) species and risk factors associated in natural infecting calves at the Southern Agreste Microregion in the State of Pernambuco, Brazil

**DOI:** 10.1590/S1984-29612022026

**Published:** 2022-05-18

**Authors:** Karlla Keyla Ferreira dos Santos, Lucia Oliveira de Macedo, Ângela Imperiano da Conceição, Lucas Azevedo dos Santos, Carla Lopes de Mendonça, Leucio Câmara Alves, Rafael Antonio Nascimento Ramos, Gílcia Aparecida de Carvalho

**Affiliations:** 1 Programa de Pós-graduação em Sanidade e Reprodução de Animais de Produção, Universidade Federal Rural de Pernambuco – UFRPE, Garanhuns, PE, Brasil; 2 Laboratório de Parasitologia, Universidade Federal do Agreste de Pernambuco – UFAPE, Garanhuns, PE, Brasil; 3 Clínica de Bovinos de Garanhuns, Universidade Federal Rural de Pernambuco – UFRPE, Garanhuns, PE, Brasil; 4 Departamento de Medicina Veterinária, Universidade Federal Rural de Pernambuco – UFRPE, Recife, PE, Brasil

**Keywords:** Eimeriosis, Eimeria zuernii, cattle-rearing, coccidia, natural infection, Eimeriose, Eimeria zuernii, bovinocultura, coccídios, infecção natural

## Abstract

*Eimeria* species have importance to calves because of the economic losses. The aim of this study was to identify the species of *Eimeria* that affect calves and the risk factors associated with its natural infection. Fecal samples (387) were collected from dairy farms in the southern Agreste of Pernambuco. The feces were evaluated using the Gordon & Whitlock technique and were cultured in 2.5% potassium dichromate for sporulation of oocysts. *Odds ratio* (*OR*) were calculated to assess risk factors. *Eimeria* spp. were detected in 50.65% (196/387) of the samples. Eleven species were identified, being *Eimeria bovis* (26.64%; 548/2057), *Eimeria zuernii* (19.69%; 405/2057) and *Eimeria ellipsoidalis* (14.49%; 298/2057) those more frequent. Small herds (*OR* = 1.93), calves aged up to six months (*OR* = 2.12), absence of manure pit (*OR* = 7.52), fortnightly cleaning (*OR* = 4.71), collective calf pens (*OR* = 3.26), manual milking (*OR* = 2.16) and absence of veterinary care (*OR* = 2.28) were considered to be risk factors. The data revealed pathogenic species in more than 50% of the farms. Thus, the importance of adopting sanitary measures to reduce the spread of these protozoa in herds should be done, because of economic losses associated with its infection.

## Resumo

Espécies do gênero *Eimeria* possuem grande importância para os bezerros devido às perdas econômicas. O objetivo deste estudo foi identificar as espécies de *Eimeria* que acometem bezerros e os fatores de risco associados à infecção. Amostras fecais (387) foram coletadas de animais de fazendas leiteiras do Agreste Meridional de Pernambuco. As fezes foram avaliadas pela técnica de Gordon & Whitlock e realizada cultura em dicromato de potássio 2,5% para esporulação dos oocistos. *Odds ratio* (*OR*) foi calculada para avaliar os fatores de risco. *Eimeria* spp. foram detectadas em 50,65% (196/387) das amostras analisadas. Foram identificadas onze espécies, sendo *E. bovis* (26,64%; 548/2057), *E. zuernii* (19,69%; 405/2057) e *E. ellipsoidalis* (14,49%; 298/2057) as mais frequentes. Pequenos rebanhos (*OR* = 1,93), bezerros com idade de até seis meses (*OR* = 2,12), ausência de esterqueira (*OR* = 7,52), limpeza quinzenal (*OR* = 4,71), bezerreiros coletivos (*OR* = 3,26), ordenha manual (*OR* = 2,16) e ausência de assistência veterinária (*OR* = 2,28) foram considerados fatores de risco. Os dados revelaram espécies patogênicas em mais de 50% das propriedades. Dessa forma, ressalta-se a importância da adoção de medidas sanitárias, visando à diminuição da propagação desses protozoários nos rebanhos, e perdas econômicas associadas à infecção.

## 1. Introduction

Parasites of the genus *Eimeria* (Apicomplexa: Eimeriidae) infect domestic ruminants worldwide and are one of the main causes of diarrhea in these animals (Bangoura & Bardsley, 2020). These protozoa commonly cause significant economic losses, such as decreased productivity, developmental delay and high mortality rates (Bangoura & Daugschies, 2018).

Young animals, aged between three weeks and six months, are the most susceptible, but adult animals can also be affected and become a source of infection (Lopez-Osorio et al., 2020). Infected animals shed oocysts into the environment through feces, which under optimal conditions become sporulated between 2 to 3 days, and can then contaminate water and food. If other animals ingest this contaminated water and food, they will in turn become infected (Worku et al., 2019; Bangoura & Bardsley, 2020). Eimeriosis can induce a wide variety of clinical signs, such as diarrhea, dehydration, loss of appetite, emaciation and apathy, and can even lead to death (Cruvinel et al., 2018a; Bangoura & Bardsley, 2020). The development and severity of these signs may vary depending on the species involved, length of exposure, infecting dose and host immune status (Worku et al., 2019; Lopez-Osorio et al., 2020).

It is known that several species of *Eimeria* can infect domestic ruminants (*e.g.* sheep, goats, cattle and buffaloes). These protozoa are specific to their hosts, for example, species that affect cattle do not infect other ruminants, except for buffaloes that can be infected by *Eimeria* species there are common to cattle (Cardim et al., 2018; El-Alfy et al., 2019; Macedo et al., 2019; Bangoura & Bardsley, 2020). The diagnosis of infection with the parasite is mostly made through coproparasitological methods for detection of oocysts. However, species differentiation is challenging because this requires precise morphometric analysis (Florião et al., 2016).

Worldwide, twenty species of *Eimeria* have been identified in cattle (Kim et al., 2018). However, only fourteen species have been recorded in Brazil. Among these, the ones that are the most pathogenic are *Eimeria bovis*, *Eimeria zuernii* and *Eimeria alabamensis*. These species are associated with the development of clinical signs, whereas *E. alabamensis* is mainly associated with clinical cases in animals kept in pastures and is present with high frequency in European countries (Daugschies & Najdrowski 2005; Enemark et al., 2013; Florião et al., 2016). In addition, neurological signs may be observed concomitantly in animals that are highly parasitized by *E. zuernii* and/or *E. bovis* (Oliveira et al., 2009; Bangoura & Bardsley, 2020).

Epidemiological studies in which species infecting cattle were identified have been conducted (Lopez-Osorio et al., 2020; Macedo et al., 2020; Ola-Fadunsin et al., 2020). However, in some regions where cattle-rearing is a significant activity, these data are absent. For example, in Pernambuco, Brazil, there are no records of the species that infect these animals, or the factors that might influence infection. Only studies on sheep and goats have been conducted (Tembue et al., 2009; Macedo et al., 2019, 2020). Therefore, the aim of the present study was to investigate the diversity of *Eimeria* species that affect calves naturally, and to identify the risk factors associated with its infection at the Southern Agreste Microregion in the State of Pernambuco.

## 2. Material and Methods

### 2.1. Study area and ethical issues

**Figure 1:**
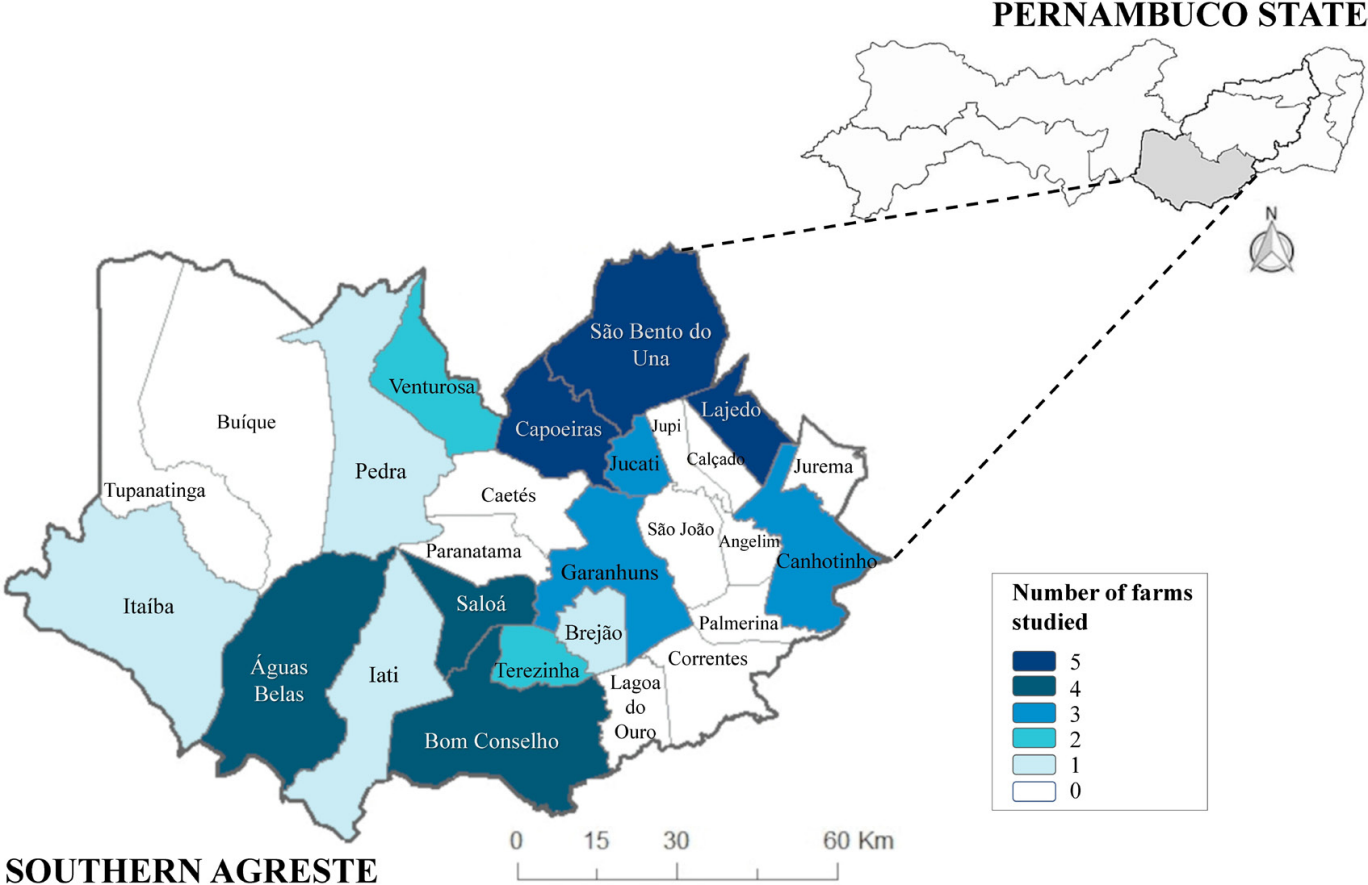
Map with the number of farms sampled at the Southern Agreste Microregion of Pernambuco, Brazil.

A cross-sectional study was conducted on 44 dairy farms in 15 municipalities located in the Southern Agreste Microregion (8°53′25” S and 36°29′34” W) in the state of Pernambuco, Brazil (Figure 1). This microregion has a predominantly hot and semi-arid climate, characterized by scarcity and irregular distribution of rainfall. It generally has average rainfall of 130 mm, temperature of 26 °C and relative air humidity of 82.5%, with a rainy season concentrated in the months of March to July.

The municipalities located in the southern Agreste microregion forms the dairy basin and are responsible for more than 80% of the total milk production of the State. This activity generates employment and income for hundreds of families, who are dependent on this. Thus, dairy cattle-rearing can be characterized as extremely important for the region (Brasil, 2011).

The present study was approved by the Ethics Committee for Animal Use of the Federal Rural University of Pernambuco (CEUA/UFRPE), under license no. 088/2019.

### 2.2. Animals studied and sample collection

The minimum sample size (n = 384) was calculated considering a cattle population of 365,428 head (IBGE, 2017) and taking the expected prevalence of *Eimeria* spp. infection to be 50%, with a 95% confidence level and statistical error of 5% (Thrusfield, 2004). The farms were selected randomly for convenience (Reis, 2003).

Between August 2019 and March 2020, fecal samples were collected directly from the rectal ampoule of 387 calves. The samples analyzed came from animals aged between one and six months (n = 349) and seven to twelve months (n = 38), without distinction of breed or gender, from dairy farms with either a semi-intensive (n = 38) or an extensive (n = 6) rearing systems.

On farms that had a semi-intensive rearing system, the animals were kept on pasture and their diet was supplemented with concentrate and mineral salt, which were supplied in troughs shortly after milking. On the other hand, animals managed through an extensive system were kept exclusively on pasture. Regardless of the breeding system, on 27 farms the milking was manual with calf at the foot, in facilities with a beaten-earth floor and collective calf pens. The animals had free access to water, which was sourced from wells and reservoirs.

To analyze risk factors, an investigative questionnaire that asked about the herd and sanitary conditions was applied to the farmers. All ethical guidelines for research, with respect for the confidentiality of the interviewees were followed. The interviewees read and signed a free and informed consent statement that had previously been approved by the CEUA.

### 2.3. Processing of samples and identification of Eimeria species

The fecal samples were analyzed by means of the flotation technique of Gordon & Whitlock (1939), to obtain oocyst counts per gram of feces. All the samples were subjected to culturing for oocyst sporulation. Regardless of calf age, for the culturing, the samples from each farm were classified into two groups (asymptomatic and symptomatic animals), depending on the absence or presence of clinical signs suggestive of eimeriosis, and were pooled. Subsequently, 4 g of feces from each pool were homogenized with 40 mL of 2.5% potassium dichromate solution ( ${\text{K}}_{2}{\text{Cr}}_{2}{\text{O}}_{7}$ ) and transferred to Petri dishes in duplicate. These were then incubated at 27 °C for a period of seven days (Duszynski & Wilber, 1997). After sporulation, the material was transferred to test tubes and subjected to centrifugation-flotation at 200 G for fifteen minutes. The supernatant was evaluated under an optical microscope (10X and 40X), with a micrometric eyepiece coupled to it, to investigate and measure the sporulated oocysts of *Eimeria* spp. (Hendrix & Robinson, 2012).

All the oocysts found in each pool were measured using the AxioVision software (version 4.8). The oocysts were identified based on morphological characteristics, as described by Daugschies & Najdrowski (2005), and by means of the dichotomous key proposed by Florião et al. (2016).

### 2.4. Data analysis

Descriptive statistics were used to obtain absolute and relative frequencies. The chi-square test with Yates correction (χ^2^) was used to compare the occurrences of infection due to different species of *Eimeria* in the rearing systems, and in different age groups of calves. The risk factors associated with *Eimeria* spp. infection were evaluated by means of univariate analysis on the variables of interest and through logistic regression analysis, taking the dependent variable to be the results from the method used (Gordon & Whitlock technique). *Odds ratio* (*OR*) values were obtained for each parameter evaluated. The significance level was taken to be 5% and all statistical analyses were performed using the BioEstat 5.3 software (Ayres et al., 2007).

## 3. Results

*Eimeria* spp. oocysts were detected in 50.65% (196/387) of the samples analyzed. These oocysts were found predominantly in animals aged one to six months (93.37%; 183/196) (χ^2^ = 3.8540; p = 0.0496). On the other hand, there was no significant difference in the rate of occurrence of infection between the different rearing systems (χ2= 0.0140; p = 0.9052), although 78.57% (154/196) of the positive samples came from animals raised semi-intensively. The morbidity rate observed was 12.76% (25/196): these animals came from 11.36% (5/44) of the farms and presented at least one clinical sign suggestive of *Eimeria* spp. infection, such as diarrhea, developmental delay, bristling fur and/or apathy.

**Figure 2:**
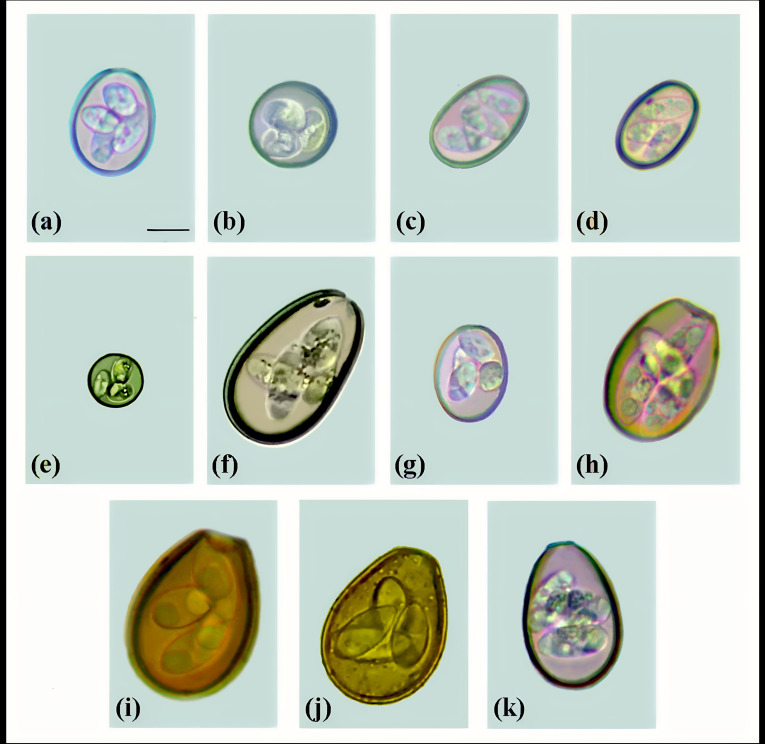
*Eimeria* species identified at the Southern Agreste Microregion of Pernambuco. (a) *Eimeria bovis*; (b) *Eimeria zuernii*; (c) *Eimeria ellipsoidalis*; (d) *Eimeria cylindrica*; (e) *Eimeria subspherica*; (f) *Eimeria auburnensis*; (g) *Eimeria alabamensis*; (h) *Eimeria canadensis*; (i) *Eimeria bukidnonensis*; (j) *Eimeria wyomingensis*; (k) *Eimeria brasiliensis*. (Scale bar: 10µm).

Eleven species of *Eimeria* (Figure 2) were identified on 63.63% (28/44) of the farms studied. On 78.57% (22/28) of the farms, coinfection by two to eight species of *Eimeria* were detected; while in the remaining 21.43% (6/28), a single infection was observed. The number of oocysts detected in the cultures ranged from 10 to 158 oocysts per sample analyzed, totaling 2057 oocysts. Among the species of *Eimeria*, *E. bovis* (26.64%; 548/2057) was predominant, followed by *E. zuernii* (19.69%; 405/2057) and *E. ellipsoidalis* (14.54%; 299/2057) (Table 1). The species *E. bovis* and *E. zuernii* were present on 78.57% (22/28) and 53.57% (15/28) of the farms, respectively.

**Table 1:** Species morphometry, indicating mean, Upper limit (Ul) and Lower limit (Ll), of *Eimeria* oocysts and sporocysts in natural infecting calves at the Southern Agreste Microregion in the State of Pernambuco, Brazil.

**Species**	**Number** a **(%)**	**Oocyst (µm)**					**Sporocysts (µm)**	
		**Lenght**	**Width**	**Shape**	**Micropyle**	**Polar cap**	**Lenght**	**Width**
***Eimeria bovis***	548 (26.64)	28.09	21.01	Ovoid	Present	Absent	13.75	6.68
		(24-37)	(18-26)				(10-21)	(5-9)
***Eimeria zuernii***	405 (19.69)	19.84	17.38	Spherical	Absent	Absent	9.06	5.35
		(15-25)	(12-23)				(6-14)	(4-8)
***Eimeria ellipsoidalis***	299 (14.54)	23.06	15.19	Ellipsoid	Absent	Absent	11.67	5.35
		(18-28)	(13-17)				(9-16)	(4-7)
***Eimeria cylindrica***	279 (13.56)	23.36	14.94	Ellipsoid	Absent	Absent	11.51	4.68
		(20-26)	(13-18)				(10-16)	(3-6)
***Eimeria subspherica***	207 (10.06)	13.72	13.04	Spherical	Absent	Absent	6.06	3.83
		(10-15)	(10-15)				(4-10)	(3-5)
***Eimeria auburnensis***	142 (6.90)	39.89	24.91	Ovoid	Present	Absent	18.39	8.06
		(31-46)	(20-29)				(14-24)	(4-10)
***Eimeria alabamensis***	83 (4.04)	20.29	15.49	Ovoid	Absent	Absent	11.08	4.73
		(18 -23)	(9-18)				(8-14)	(3-6)
***Eimeria canadensis***	53 (2.58)	30.39	22.73	Ovoid	Present	Absent	17.27	7.58
		(26-37)	(20-27)				(14-22)	(6-9)
***Eimeria bukidnonensis***	20 (0.97)	39.33	28.33	Piriformis	Present	Absent	19.33	7.67
		(38-40)	(28-29)				(19-20)	(7-8)
***Eimeria wyomingensis***	20 (0.97)	38.17	29.33	Piriformis	Present	Absent	17.67	8.33
		(37-40)	(29-30)				(16-19)	(8-9)
***Eimeria brasiliensis*** b	1 (0.05)	34	22	Ovoid	Present	Present	17	8

a based in 2057 oocysts measured;

b Upper limit (Ul) – Lower limit (Ll) were not determined, because only one oocyst of this species was observed in this study.

The symptomatic animals were coinfected by two or more species, and *E. bovis* (53.69%; 262/488) was the most abundant. *E. zuernii* (19.26%; 94/488), *E. ellipsoidalis* (13.93%; 68/488), *Eimeria cylindrica* (7.38%; 36/488), *Eimeria auburnensis* (4.92%; 24/488) and *Eimeria subspherica* (0.82%; 4/488) were also found in these animals. The number of sporulated oocysts per sample analyzed ranged from 60 to 172.

The univariate analysis showed that the following were risk factors for infection by *Eimeria* spp.: calves up to 6 months of age, small herds (≤ 50 animals), absence of a manure pit, collective calf pens, long periodicity of cleaning of facilities, absence of veterinary care and manual milking (Table 2).

**Table 2:** Risk factors associated with natural infection by *Eimeria* spp. in calves at the Southern Agreste Microregion in the State of Pernambuco, Brazil.

**Variables**	**N**	**positive calves**	***OR* (CI 95%)**	**p-values**
**Rearing system**				
Extensive	81	42 (51.85%)	1.06 (0.65 – 1.74)	0.9829
Semi-intensive	306	154 (50.33%)		
**Age (months)**				
≤6	349	183 (52.44%)	2.12 (1.05 – 4.28)	0.0496*
>6	38	13 (34.21%)		
**Herd size (animals)**				
≤50	118	73 (61.86%)	1.93 (1.24 – 2.99)	0.0049*
51 to 150	111	62 (55.86%)	1.34 (0.86 – 2.09)	0.2350
> 150	158	61 (38.61%)		
**Manure pit**				
Yes	23	03 (13.04%)		
No	364	193 (53.02%)	7.52 (2.19 – 25.76)	0.0005*
**Collective calf**				
Yes	367	191 (52.04%)	3.25 (1.15 – 9.14)	0.0335*
No	20	05 (25.00%)		
**Periodicity of cleaning**				
Daily	35	05 (14.29%)		
Weekly	118	63 (53.39%)	1.17 (0.75 – 1.81)	0.5454
Biweekly	59	47 (79.66%)	4.71 (2.41 – 9.19)	<0.0001*
Monthly	175	81 (46.28%)	0.73 (0.49 – 1.09)	0.1452
**Veterinary assistance**				
Yes	136	51 (37.50%)		
No	251	145 (57.77%)	2.28 (1.49 – 3.49)	0.0002*
**Milking type**				
Mechanical	204	85 (41.67%)		
Manual	185	111 (60.66%)	2.16 (1.44 – 3.24)	0.0003*

N: Total samples; *OR: Odds ratio*; CI: Confidence interval; * p <0.05 significant association.

## 4. Discussion

This study evaluated the diversity of *Eimeria* species that infect calves in the State of Pernambuco. The data indicated that the animals living in this region are parasitized by a wide variety of species of *Eimeria* (11 species) (Figure 2). The diversity of species differs according to the region of Brazil, as demonstrated in other studies (Vidal et al., 2013; Cruvinel et al., 2018b). On average, the number of species infecting cattle ranges from six to twelve (Cruvinel et al., 2018b, 2020). The frequency of positivity among the animals of the present study (50.65%) was similar to that of a previous report from northeastern Brazil, in which positivity of 51.22% was detected among calves less than one year of age (Almeida et al., 2011). In different regions of Brazil, the prevalence of *Eimeria* spp. infection in cattle herds has been found to range from 3.46% to 98.10%. This diversity was probably due to factors related to management, climatic conditions, host age and intensity of infection (Vidal et al., 2013; Tomczuk et al., 2015).

Several risk factors associated with *Eimeria* spp. infection have been identified in cattle (Worku et al., 2019; Cruvinel et al., 2020; Lopez-Osorio et al., 2020). In the present study, animals less than six months old were the most parasitized. Among these animals, 25 calves showed some clinical sign suggestive of *Eimeria* spp. infection and, in cultures, they presented parasite loads of pathogenic species that were larger than the loads of asymptomatic calves. Previous exposure to coccidia has been found to promote development of partial immunity, thus resulting in elimination of oocysts in smaller amounts (Bangoura & Bardsley, 2020). Development of clinical signs depends on several factors, such as immunosuppression, stress, exposure to high doses of sporulated oocysts and concomitant diseases (Worku et al., 2019; Ola-Fadunsin et al., 2020).

However, the presence of diarrhea may be related to gastrintestinal disorders of several origins, such as nutritional, bacterial, parasitic and viral. Thus, several etiological agents may be involved (Pomim et al., 2021).

Because young animals have immature immune systems, they are more susceptible to the clinical manifestations of eimeriosis and to the consequences resulting from the subclinical form. These events can have a negative impact on herd productivity through delayed growth among the animals affected (Enemark et al., 2013; Worku et al., 2019; Lopez-Osorio et al., 2020). On the other hand, infected calves older than six months of age act as source of infection for younger calves, and normally they do not exhibit clinical signs (Lopez-Osorio et al., 2020).

Although no significant difference in positivity was observed between the different types of rearing system, 78.57% of the positive samples came from farms that were using a semi-intensive rearing system. This finding may be related to the management practices adopted, such as confinement of calves in collective facilities, because agglomeration of animals favors greater environmental contamination (Worku et al., 2019). Although calves living in intensive management systems are affected with greater severity (Ferraz et al., 2018), higher occurrence has also been detected among calves that are reared collectively on pasture, due to the ease of contamination of pastures and water sources by oocysts (Cruvinel et al., 2018a).

It is known that larger herds are more affected by eimeriosis due to high animal density, especially those reared under intensive management (Lopez-Osorio et al., 2020). However, in the present study, smaller herds (< 50 animals) were identified as a risk factor (*OR* = 1.93; p = 0.0049). This may be related to inadequate management and hygiene practices adopted by small producers who may not have access to correct information, such as with regard to animal agglomeration and low frequency of cleaning, which thus gives rise to high accumulation of feces and greater infection rates (Gonçalves et al., 2014). Another important factor was the lack of veterinary care on the farms studied (*OR* = 2.28; p = 0.0002). In a previous study, the presence of a veterinarian on the farm was taken to be a protective factor for the animals (Lopez-Osorio et al., 2020). Thus, the presence of technical assistance on the farm prevents the emergence of several diseases in the herd, which contributes to better performance regarding milk production (Gonçalves et al., 2014).

Absence of a manure pit (*OR* = 7.52; p = 0.0005) and long periodicity of cleaning of the facilities (*OR* = 4.71; p < 0.0001) are risk factors related to hygiene. These, together with management factors such as the use of collective calf pens (*OR* = 8.52; p = 0.0391) and manual milking (*OR* = 2.16; p = 0.0003), may have contributed to the maintenance and propagation of the protozoa in these herds. Calves kept in collective facilities and under poor hygiene conditions are more likely to develop eimeriosis than are calves kept in individual facilities and under good hygiene conditions (Worku et al., 2019). Manual milking with calves at the foot is a common practice among the farmers in the region of the present study. Thus, these animals are more exposed to oocysts, since adult animals are a source of infection. This suggests that keeping the calves with adults favors higher infection rates, due to the higher chance of ingestion of a large number of oocysts (Cruvinel et al., 2018a; Worku et al., 2019).

In the present study, the predominant species were *E. bovis* and *E. zuernii*. Their presence indicates that there is a high risk of clinical eimeriosis in the herds of the region. The species *E. bovis* is the one most frequently found in Brazil and this species, together with *E. zuernii*, are the ones that are most pathogenic, once that are responsible for causing moderate to severe enteritis, which can lead to the death of animals (Enemark et al., 2013; Cardim et al., 2018; Bangoura & Bardsley, 2020). In addition, *E. zuernii* can cause neurological changes, which may occur either with or without associated enteric signs. Presence of these changes can lead to death because they are confounded with other pathological conditions that causes similar nervous signs (Oliveira et al., 2009). Clinical signs suggestive of *Eimeria* spp. infection were present in 12.76% of the calves evaluated: these were infected by pathogenic species (*E. bovis* and *E. zuernii*), but none of the animals infected by *E. zuernii* had neurological signs. Nonetheless, this does not rule out the possibility that calves in this region might be affected by this clinical picture of the disease.

Although studies related to bovine eimeriosis in this region have not been previously conducted, veterinarians working in these municipalities have reported the presence of calves affected by the disease, as well as the resultant consequences in herds, especially with regard to the high mortality rate. Although *E. ellipsoidalis* was one of the species most detected (14.54%), it is known that there is no relationship between presence of this species and development of severe clinical signs of eimeriosis in infected animals. This species is associated with the subclinical form of the disease, considering that it has low pathogenicity (Bangoura & Bardsley, 2020). Thus, it can be suggested that dairy herds in this region are prone to the long-term consequences of *Eimeria* infection, such as reduced animal performance due to fertility delay, caused by decreased weight gain and lower final weight (Bangoura & Daugschies, 2018; Bangoura & Bardsley, 2020; Lopez-Osorio et al., 2020). However, further studies aimed at evaluating the real economic impact of subclinical eimeriosis in cattle herds in this region should be conducted in order to better elucidate these facts.

On the other hand, *E. auburnensis* and *Eimeria alabamensis* were found less frequently, but no less importantly. These species are seen as potential strains causing the clinical form of the disease, especially *E. alabamensis*, which has been an important cause of the occurrence of clinical cases in several countries, such as Germany, Netherlands, and Sweden, where it occurs more frequently, representing a clinical problem in animals that are raised extensively (Daugschies & Najdrowski 2005; Rijpert-Duvivier et al., 2021). Because it has a short prepatent period (6 to 11 days), outbreaks can be observed soon after new animals are introduced to a highly contaminated pasture. Mortality is generally low and occurs mainly due to co-infections with pathogenic species. In addition, dehydration and growth retardation can be observed in calves with single *E. alabamensis* infections (Daugschies & Najdrowski 2005; Bangoura & Bardsley, 2020).

Coinfections were observed on most farms (78.57%), thus emphasizing the fact that animals in the region studied are prone to develop eimeriosis, since coinfection can result in the onset of diarrhea, especially if two or more pathogenic species are present (Bangoura & Daugschies, 2018; Kim et al., 2018).

This study was the first to identify *Eimeria* species and to evaluate the risk factors associated with infection among calves in the southern Agreste region of Pernambuco.

In conclusion, the data obtained clarified factors that influences *Eimeria* spp. infection in calves. This study revealed that the low level of sanitary management of dairy farms in this region may have contributed to the appearance of cases of eimeriosis in these herds. Thus, it is important to highlight the need for development of sanitary strategies aimed at reducing the spread of these agents in cattle herds, since economic losses are associated not only with the immediate damage caused by the infection, but also with diminished performance of the herd over a long term.

## References

[B001] Almeida VA, Magalhães VCS, Muniz Neta ES, Munhoz AD (2011). Frequency of species of the Genus *Eimeria* in naturally infected cattle in Southern Bahia, Northeast Brazil. Braz J Vet Parasitol.

[B002] Ayres M, Ayres M, Ayres DL, Santos AA (2007). BioEstat – statistical applications on the biomedical sciences..

[B003] Bangoura B, Daugschies A, Florin-Christensen M, Schnittger L (2018). Parasitic protozoa of farm animals and pets..

[B004] Bangoura B, Bardsley KD (2020). Ruminant Coccidiosis. Vet Clin Food Anim Pract.

[B005] Brasil (2011). Plano Territorial de Desenvolvimento Rural Sustentável do Agreste Meridional de Pernambuco..

[B006] Cardim ST, Seixas M, Tabacow VBD, Taroda A, Carneiro PG, Martins TA (2018). Prevalence of *Eimeria* spp. in calves from dairy farms in northern Paraná State, Brazil. Braz J Vet Parasitol.

[B007] Cruvinel LB, Bastos TSA, Nicaretta JE, Couto LFM, Borges DGL, Borges FA (2018). Surtos consecutivos ocasionados por *Eimeria zuernii* em bezerros de corte de uma propriedade do estado de São Paulo. Pesq Vet Bras.

[B008] Cruvinel LB, Nicaretta JE, Bastos TSA, Couto LFM, Santos JB, Zapa DMB (2018). *Eimeria* species in dairy and beef cattle of different ages in Goiás state, Brazil. Braz J Vet Parasitol.

[B009] Cruvinel LB, Ayres H, Zapa DMB, Nicaretta JE, Couto LFM, Heller LM (2020). Prevalence and risk factors for agents causing diarrhea (Coronavirus, Rotavirus, *Cryptosporidium* spp., *Eimeria* spp. and nematodes helminthes) according to age in dairy calves from Brazil. Trop Anim Health Prod.

[B010] Daugschies A, Najdrowski M (2005). Eimeriosis in cattle: current Understanding. J Vet Med B Infect Dis Vet Public Health.

[B011] Duszynski DW, Wilber PG (1997). A guideline for the preparation of species descriptions in the Eimeriidae. J Parasitol.

[B012] El-Alfy E, Abbas IE, Al-Kappany Y, Al-Araby M, Abu-Elwafa SA, Dubey JP (2019). Prevalence of *Eimeria* species in water buffaloes (*Bubalus bubalis*) from Egypt and first report of *Eimeria bareillyi* oocysts. J Parasitol.

[B013] Enemark HL, Dahl J, Enemark JMD (2013). Eimeriosis in Danish Dairy Calves – correlation between species, oocyst excretion and diarrhoea. Parasitol Res.

[B014] Ferraz A, Santos EM, Castro TA, Dallmann PRJ, Pinto DM, Nizole LQ (2018). Ocorrência de parasitos gastrintestinais diagnosticados em bovinos pelo laboratório de doenças parasitárias da Universidade Federal de Pelotas (Brasil), nos anos de 2015 a 2017. Vet Foco.

[B015] Florião MM, Lopes BB, Berto BP, Lopes CWG (2016). New approaches for morphological diagnosis of bovine *Eimeria* species: a study on a subtropical organic dairy farm in Brazil. Trop Anim Health Prod.

[B016] Gonçalves ACS, Roma LC, Fonseca MI, Nadruz BV, Bürger KP, Rossi GAM (2014). Assistência técnica e extensão rural: sua importância para a melhoria da produção leiteira – Relato de caso. Rev Bras Hig Sanid Anim.

[B017] Gordon HM, Whitlock HV (1939). A new technique for counting nematode eggs in sheep faeces. J Counc Sci Ind Res.

[B018] Hendrix CM, Robinson E (2012). Diagnostic parasitology for veterinary technicians..

[B019] IBGE (2017). Censo Agropecuário 2017: Resultados definitivos.

[B020] Kim HC, Choe C, Kim S, Chae JS, Yu DH, Park J (2018). Epidemiological survey on *Eimeria* spp. associated with diarrhea in pre-weaned native korean calves. Korean J Parasitol.

[B021] Lopez-Osorio S, Villar D, Failing K, Taubert A, Hermosilla C, Chaparro-Gutierrez JJ (2020). Epidemiological survey and risk factor analysis on *Eimeria* infections in calves and young cattle up to 1 year old in Colombia. Parasitol Res.

[B022] Macedo LO, Santos MAB, Silva NMM, Barros GMMR, Alves LC, Giannelli A (2019). Morphological and epidemiological data on *Eimeria* species infecting small ruminants in Brazil. Small Rumin Res.

[B023] Macedo LO, Bezerra-Santos MA, Mendonça CL, Alves LC, Ramos RAN, Carvalho GA (2020). Prevalence and risk factors associated with infection by *Eimeria* spp. in goats and sheep in Northeastern Brazil. J Parasit Dis.

[B024] Ola-Fadunsin SD, Rabiu M, Hussain K, Sanda IM, Ganiyu IA (2020). Epidemiological studies of *Eimeria* species of cattle in Ilorin, North-Central Nigeria. Ann Parasitol.

[B025] Oliveira PCL, Sampaio RL, Lacerda MS, Alvarenga RR, Espinoza MF (2009). Coccidiose entérica, associada à encefalopatia, em vaca Gir adulta (Relato de caso). Cienc Anim Bras.

[B026] Pomim GP, Neves PMS, Silva RAB, Garcia MS, Carvalho GF, Melo AF (2021). Perfil do conhecimento de produtores sobre diarreia neonatal bovina e seu impacto para a atividade. Rev Bras Hig Sanid Anim.

[B027] Reis JC (2003). Estatística aplicada à pesquisa em ciência veterinária..

[B028] Rijpert-Duvivier ACM, Geurts CPH, Vangroenweghe F, Allais L, van Doorn DCK (2021). Oocyst shedding patterns of *Eimeria* species and their association with management and performance at ten rose veal started farms in the Netherlands. Vet Parasitol Reg Stud Reports.

[B029] Tembue AASM, Ramos RAN, Lima MM, Faustino MAG, Meunier IMJ, Alvez LC (2009). Espécies do gênero *Eimeria* Schneider, 1875 (Apicomplexa: Eimeriidae) em pequenos ruminantes, provenientes do município de Ibimirim, estado de Pernambuco. Vet Not.

[B030] Thrusfield M (2004). Epidemiologia veterinária..

[B031] Tomczuk K, Grzybek M, Szczepaniak K, Studzinska M, Demkowska-Kutrzepa M, Roczén-Karczmarz M (2015). Analysis of intrinsic and extrinsic factors influencing the dynamics of bovine *Eimeria* spp. from central-eastern Poland. Vet Parasitol.

[B032] Vidal LGP, Fagundes TF, Pantoja CS, Menezes RCAA (2013). Morfometria de oocistos de *Eimeria* em bezerras segundo a faixa etária e a intensidade infecção, Município de Piraí, RJ. Rev Bras Saúde Prod Anim.

[B033] Worku K, Hamid M, Dubie T (2019). Study on prevalence and risk factors of calf coccidiosis in around Sekota town, Northern Ethiopia. Int J Curr Res Biol Med.

